# Isolation and Characterization of *Vibrio kanaloae* as a Major Pathogen Associated with Mass Mortalities of Ark Clam, *Scapharca broughtonii*, in Cold Season

**DOI:** 10.3390/microorganisms9102161

**Published:** 2021-10-16

**Authors:** Bowen Huang, Xiang Zhang, Chongming Wang, Changming Bai, Chen Li, Chenghua Li, Lusheng Xin

**Affiliations:** 1 School of Marine Sciences, Ningbo University, Ningbo 315211, China; huangbwn@163.com; 2 Key Laboratory of Marine Aquaculture Disease Control, Ministry of Agriculture, Qingdao Key Laboratory of Marine Aquaculture Epidemiology and Biosecurity, Yellow Sea Fisheries Research Institute, Chinese Academy of Fishery Sciences, Qingdao 266071, China; zhxiang1997@126.com (X.Z.); wangcm@ysfri.ac.cn (C.W.); baicm@ysfri.ac.cn (C.B.); lichen@ysfri.ac.cn (C.L.)

**Keywords:** *Vibrio kanaloae*, *Scapharca broughtonii*, low temperature, hemolytic activity, siderophore production

## Abstract

High temperature is a risk factor for vibriosis outbreaks. Most vibrios are opportunistic pathogens that cause the mortality of aquatic animals at the *vibrio* optimal growth temperature (~25 °C), whereas a dominant *Vibrio kanaloae* strain SbA1-1 is isolated from natural diseased ark clams (*Scapharca broughtonii*) during cold seasons in this study. Consistent symptoms and histopathological features reappeared under an immersion infection with SbA1-1 performed at 15 °C. The pathogenicity difference of SbA1-1 was assessed under different temperatures (15 °C and 25 °C). The cumulative mortality rates of ark clams were significantly higher at the low temperature (15 °C) than at the high temperature (25 °C); up to 98% on 16th day post SbA1-1 infection. While the growth ratio of SbA1-1 was retarded at the low temperature, the hemolytic activity and siderophores productivity of SbA1-1 were increased. This study constitutes the first isolation of *V. kanaloae* from the natural diseased ark clams (*S. broughtonii*) in cold seasons and the exposition of the dissimilar pathogenicity of SbA1-1 at a different temperature. All the above indicates that *V. kanaloae* constitutes a threat to ark clam culture, especially in cold seasons.

## 1. Introduction

Ark clams (blood clam), *Scapharca broughtonii*, are members of the Arcoidea superfamily, class Bivalvia, phylum Mollusca [1]. It is an important commercial shellfish in North Asian countries [2,3]. However, due to excessive aquaculture and habitat deterioration, ark clams are susceptible to pathogen infection and have experienced mass mortalities [4,5,6,7]. Since 2012, epidemiological investigations have confirmed that the ark clam is the host of the Ostreid herpesvirus 1 (OsHV-1) and OsHV-1 is considered a major virus pathogen associated with the mass mortality of molluscs during the warm seasons, leading to severe economic losses [6,8]. Moreover, in 2017, the bacterial pathogen, *Vibrio harveyi*, was found to cause the mass mortality of ark clams [4].

The occurrence of an aquatic disease is a comprehensive interaction of hosts, microorganisms, and environmental factors [9,10]. Temperature is one of the most common environmental factors that could trigger dynamic changes in the growth, metabolic process and pathogenic capacity of bacteria [11,12]. Vibriosis, caused by *Vibrio* spp., is one of the most prevalent bacterial diseases in the cultured mollusc worldwide [13,14,15,16,17,18]. Aside from the well-known opportunistic pathogens associated with mass mortality in molluscs: *V. harveyi*, *V. parahaemolyticus*, *V. splendidus*, *V. alginolyticus* [19,20,21,22], more than 50 new species of the genus *Vibrio* have been recorded in the last dozen years, most of which present in the marine environment [23]. Some of these species, such as *V. kanaloae*, *V. aestuarianus*, *V. gigantis*, and *V. lentus*, have been identified as the primary cause of mortality in diffrerent molluscs, severely affecting their wild and farmed populations [9,23,24,25,26]. The opportunistic pathogenic vibrios commonly possess a high metabolism level and proliferation rate in warm seasons, which can cause aquatic mortality [27,28,29]. Comparatively, in cold seasons, bacteria undergo a slow growth and a “latent” or “dormant” phase, while few vibrios retain pathogenicity, or even a higher virulence, by producing much more virulent factors at low temperatures [18,30,31,32,33].

Iron is an essential biocatalyst or electron carrier for almost all organisms [34,35]. For the effective colonization in hosts, bacteria must obtain host-available iron [34,36]. *Vibrio* has evolved several highly efficiency iron acquisition systems to sequester different sources of host iron. Typically, vibrios can secrete iron-chelating compounds known as siderophores to compete for the available iron with host iron-containing proteins, or employ specific outer-membrane receptors to obtain iron from free heme or heme-containing proteins released by hemolysins-cleaved cells [37,38,39]. These iron acquisition mechanisms of *Vibrio* are closely correlated with their virulence. The pathogenicity of some vibrios, such as *V. anguillarum*, *V. vulnificus*, depends largely on their ability to absorb and utilize iron [33,40,41,42].

In the present study, during our epidemiological survey, a diseased ark clam population was investigated from an aquaculture farm in winter 2020. Their main symptoms were gill erosion and foot thinning, which were consistent with previously described bacteriosis [4]. Thus, we firstly isolated potential pathogenetic bacteria from those ark clams, then explored the influence of temperature on the pathogenicity of *V. kanaloae*, aiming to provide theoretical support for the development of effective epidemiological management strategies to prevent and control *V. kanaloae* outbreaks.

## 2. Materials and Methods

### 2.1. Collection of Diseased S. broughtonii Samples

In December 2020, a diseased ark clam population was collected from an aquaculture farm in the Yellow Sea with a water temperature of approximately 15 °C. The symptoms of those ark clams included gill erosion, hepatopancreas hyperemia, foot thinness, and a slow response to external stimuli.

### 2.2. Detection of OsHV-1

Forty ark clams were randomly selected, 20 mg mantles from four random individuals were collected and mixed as a sample. Ten samples were parallelly prepared for detection. Total DNA of each sample was extracted using the TIANamp Marine Animals DNA Kit (Tiangen, Beijing, China) according to the manufacturer’s instructions. Detection of OsHV-1 was performed according to a previously described protocol based on the Taqman probe method using a pair of primers (BF and B4) and the TaqMan probe BP [43]. The reaction was performed based on Bio-Rad CFX Connect Real-Time system (Hercules, CA, USA) following 1 cycle of 95 °C for 10 min, 40 cycles of 95 °C for 10 s, 60 °C for 30 s. The quantification of OsHV-1 was calculated from the standard curve, which was generated from a 10-fold dilution series (10^8^–10^1^ copies/mL) of the plasmid containing the target sequence.

### 2.3. Histopathology Analysis of Diseased S. broughtonii

For histopathologic analysis, the tissues of ark clams were dissected and fixed in Davidson’s alcohol formalin-acetic acid fixative (DAFA) for 24 h. After dehydration and embedding in paraffin, tissue blocks were cut into 5 mm thick sections. Then, tissue sections were stained with hematoxylin and eosin (H&E) solution for observation. For tracing tissue iron, the Prussian blue histochemical iron (III) assay was performed according to the product instruction (Solarbio, Beijing, China). Tissue sections were stained in a mixture of 5% potassium ferricyanide and 5% hydrochloric acid for 30 min, washed and restained with nuclear-fast red. Gram staining was performed on tissue sections to show bacterial aggregation in the examined tissue (Solarbio).

### 2.4. Bacterial Isolation

Hepatopancreas and gills were dissected from ten diseased ark clams and homogenized in 1 mL cooled sterile seawater. Then, the homogenate was centrifuged at 800× *g*, 4 °C for 10 min, and the supernatant was serially diluted and plated on 2216E agar plates. The plates were incubated at 15 ± 2 °C for 48 h.

### 2.5. Bacteria Identification

After being incubated, twenty individual colonies were randomly selected from 2216E agar plates of each ark clam and sub-cultured for identification. Bacterial DNA was extracted using the Bacterial DNA Kit (Omega Bio-Tek, Norcross, GA, USA) following the manufacturer’s instructions. The preliminary identification of isolated bacteria was performed using the primers 27F and 1492R to amplify 16S rDNA (Table 1) [44]. In addition, the housekeeping genes *atpA* (ATP synthase alpha subunit gene), *mreB* (rod shaping protein B subunit gene), *pyrH* (uridylate kinase gene), *recA* (recombinase A gene), and *rpoA* (RNA polymerase alpha subunit gene) were amplified and sequenced, as previously described, to further confirm the species of isolated vibrios [45]. The concatenated sequences of *atpA*-*mreB*-*pyrH*-*recA*-*rpoA* from the isolates and other reported *Vibrio* strains were aligned using the CLUSTAL_W tool and the phylogenetic tree was evaluated using bootstrap analysis based on the neighbor-joining (NJ) method with 1000 replicates, which was embedded in MEGA version 7.0.

### 2.6. Morphological Structure Analysis of V. kanaloae Strain SbA1-1

After incubating SbA1-1 in 2216E medium at 15 °C for 12 h, the bacterial culture went through static settlement for 20 min; the precipitate was washed twice with sterile seawater and concentrated 10-fold with sterile seawater. For electron microscopic observation, 10 μL of the sample was placed on the grid. The grid was stained with 2% phosphotungstic acid at neutral pH and stored at room temperature before imaging. The morphological structure of SbA1-1 was observed by electron microscope (HT7700, Hitachi, Tokyo, Japan) with 5000× magnification. For light microscope morphologic analysis, bacteria were stained with Gram-Stain Kit (Solarbio) and observed under the Nikon Eclipse E80i light microscope (Nikon, Tokyo, Japan). Simultaneously, SbA1-1 were cultured on thiosulfate-citrate-bile salts-sucrose (TCBS) agar to observe the colony morphology.

### 2.7. Experimental Infection

Healthy ark clams for the experimental challenge were obtained from a local farm in Yantai, China. These ark clams were tested as OsHV-1 negative in Section 2.2. Before the experiment, the ark clams were acclimatized in tanks at 15 °C or 25 °C for two weeks. The ark clams, acclimated at different temperatures, were randomly divided into four groups: 15 °C challenge, and control groups; 25 °C challenge, and control groups (60 ark clams were equally separated and cultured in three 50 L tanks in each group as repeats). Ark clams in the challenge group were immersed in *V. kanaloae* strain SbA1-1 at a final concentration of 1 × 10^5^ CFU/mL, while no bacteria were added in the control group. The dead ark clams were counted and sampled every 12 h for 18 days.

### 2.8. Detection of V. kanaloae in Ark Clam Using Nested PCR

The gene *iucA*/*iucC* is responsible for the synthesis of siderophore participating in iron acquisition, which is an essential mechanism for the survival of bacteria in the host and also plays an important role in bacterial pathogenicity [51,52]. In this study, specific nested PCR primers were designed based on the complete sequence of the *V. kanaloae* IucA/IucC family siderophore biosynthesis protein gene (Genebank ID QPK06640.1). The external primers of the nested PCR were designed to amplify positions 93 to 1213 of the *V. kanaloae* IucA/IucC family siderophore biosynthesis protein gene fragment, and the internal primers were designed to amplify positions 209 to 457 (Table 1). The specificity of the nested PCR primers was evaluated by Primer-BLAST (https://www.ncbi.nlm.nih.gov, accessed on 14 March 2021) and verified by using genomic DNA of 12 non- *V. kanaloae* bacteria isolated from shellfish and healthy ark clam genomic DNA.

PCR reacted using KOD DNA polymerase (Toyobo, Osaka, Japan). A measure of 23 μL of PCR reaction mixture was prepared according to the manufacturer’s protocol for each 2 μL DNA sample. The 1st-step PCR was initiated using external primers at 94 °C for 30 s, followed by 30 cycles of 98 °C for 10 s, 55 °C for 5 s, and 68 °C for 30 s. The 2nd-step PCR was performed by using the internal primers and 2 μL of 10-fold, prediluted PCR amplification product as a template. The PCR cycling conditions were 94 °C for 30 s, followed by 30 cycles of 98 °C for 10 s, 58 °C for 5 s, and 68 °C for 10 s. *V. kanaloae* DNA was used as a positive control, healthy ark clam tissue DNA extracted using the TIANamp Marine Animals DNA Kit (TIANGEN) was used as a negative control, and deionized distilled water was used as a blank control. The PCR products of 10 μL were analyzed in 2% (*w/v*) agarose gels.

### 2.9. Quantitation of Vibrio Abundance

Each DNA sample was extracted and mixed from two individual tissues using the TIANamp Marine Animal DNA Kit (Tiangen) and the DNA was quantified using a spectrophotometer. Five samples were parallelly set for gill and hepatopancreas, respectively, in each experimental group. Total vibrios were assessed using SYBR Green qPCR method utilizing the specific *Vibrio* 16S rDNA specific primers (Table 1) [50]. PCR reactions were performed using THUNDERBIRD SYBR^®^ qPCR Mix (Toyobo). The *Vibrio* quantitation was calculated from the standard curve generated by *V. kanaloae* 16S rDNA sequences cloned into the pUC57 vector.

### 2.10. Minimal Inhibitory Concentration (MIC)Determination of 2,2′-Dipyridyl (DP)

To mimic the host’s iron-limiting microenvironment, 2,2′-dipyridyl (DP) (99+%, Alfa Aesar, Haverhill, MA, USA) concentration was measured as previously described [53,54]. Overnight cultures of *V. kanaloae* strain SbA1-1 were transferred into fresh 2216E medium at a ratio of 1:100, which contained DP at different concentrations of 0, 20, 40, 80, 160, 320 and 640 μM. The growth of SbA1-1 was monitored by measuring OD_600_ at 0, 6, 12, 18, 24, 36 and 48 h. The MIC of DP was determined as the lowest concentration at which no significant bacterial growth was detected.

### 2.11. Hemolysis Assay

Overnight cultures of *V. kanaloae* strain SbA1-1 were spotted onto sheep blood plates, and these plates were equally separated and incubated at 15 °C and 25 °C, respectively, for four days. Hemolytic activity was assessed by visual inspection and measurement of the hemolytic zone. Meanwhile, hemoglobin released from erythrocytes was quantified according to a previously described method with some modifications to determine the hemolytic activity of SbA1-1 at different temperatures [55]. Briefly, SbA1-1 was incubated in 2216E medium with 160 μM DP (DPM medium) at 15 °C or 25 °C. When SbA1-1 entered the stationary phase, the cells were collected by centrifugation, were resuspended (10^8^ CFU/mL) and then continued to incubate at the above two temperature conditions, respectively. For 50, 100, and 150 min incubation, the bacterial medium supernatant was collected by centrifugation at 5000× *g* for 5 min. A measure of 10 μL 1% sheep erythrocytes was added to 1 mL medium supernatant (filtered through 0.22 µm pore-size filters). At the same time, 10 μL of 1% sheep erythrocytes were added to 1 mL of DPM medium with and without 0.1% Triton-X 100 as positive and negative controls, respectively. After incubation at 37 °C for 1 h, erythrocytes were centrifuged at 800× *g* for 10 min, then 200 uL of supernatant was transferred to a 96-well plate. The release of hemoglobin was assessed by measuring OD_543_ using a Varioskan Flash microplate reader (Thermo Fisher Scientific, Vantaa, Finland). Hemolysis percentage was calculated by hemolysis (%) = (OD_s_ − OD_n_)/(OD_p_ − OD_n_) × 100%, where OD_s_ = sample absorbance, OD_n_ = negative control absorbance, and OD_p_ = positive control absorbance.

### 2.12. CAS Assay

Chromeazurol S (CAS) method was performed to determine the ability of siderophore production by *V. kanaloae* strain SbA1-1 [56]. Overnight cultures of SbA1-1 were spotted on CAS agar medium (Coolaber, Beijing, China). After parallel incubation at 15 °C and 25 °C, respectively for 6 days, siderophore production was qualitatively observed by visual inspection and measurement of the orange–yellow halos around the colonies. Meanwhile, the CAS method was also performed to quantify the siderophores production at different temperatures. A measure of 0.5 mL of supernatant (filtered through 0.22 µm pore-size filters) from each culture was mixed with equal volumes of CAS assay solution (Coolaber). The negative control was DPM medium mixed with CAS assay solution only. After 1 h incubation at room temperature, the absorbance at 630 nm was measured. The siderophore production was calculated using the formula: siderophore (%) = (OD_n_ − OD_s_)/OD_n_) × 100%, where OD_s_ = sample absorbance and OD_n_ = negative control absorbance.

### 2.13. Statistical Analysis

The statistical analysis was performed using Statistical Package for Social Sciences (SPSS). The results were expressed as mean ± standard error (S.E.), differences between the control and experimental groups were assessed using one-way ANOVA, and significance was accepted at *p* < 0.05.

## 3. Results

### 3.1. Pathological Characteristics of Natural Diseased S. broughtonii in Cold Season

**Figure 1:**
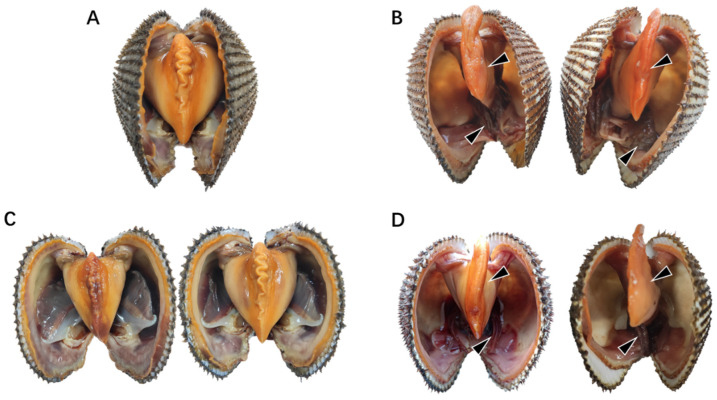
Symptoms of natural diseased ark clams. (**A**) Healthy ark clams. (**B**) Natural diseased ark clams. (**C**) Ark clams in the control group without SbA1-1 challenge at low (15 °C, **left**) and high (25 °C, **right**) temperatures. (**D**) Ark clams challenged with SbA1-1 strain (1 × 10^5^ CFU/mL) under 15 °C (**left**) and 25 °C (**right**). The black arrow marks the site of the lesion.

**Figure 2:**
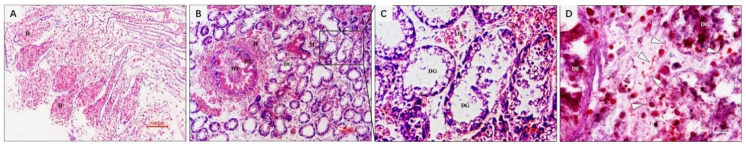
Histopathological changes of natural diseased ark clam tissues. (**A**) Eroded gill tissue with massive hemocytes infiltration. (**B**) Hepatopancreas with massive hemocytes infiltration, digestive epithelium with extensive necrosis. (**C**) Glandular tubule with erosion, severe vacuolization of epithelial cells. (**D**) Gram-negative bacteria in the connective tissue around digestive tubes and glands. H, hemocyte; DE, digestive epithelium; DT, digestive tubule; DG, digestive gland. Bar in (**A**,**B**): 100 μm; bar in C and D: 10 µm.

In natural diseased ark clams, *S. broughtonii*, the representative symptoms included severe foot thinning and gill erosion (Figure 1A,B). Firstly, the pandemic virus infection by OsHV-1 was excluded by real-time PCR using a Taqman probe-based method. The OsHV-1 copy number of the 10 tested samples was below 10 copies/ng DNA with no statistical difference from the negative control (Figure S1). HE staining showed that there were numbers of hemocytes infiltrating the gills and hepatopancreas (Figure 2A,B). The hepatopancreas presented with an extensive necrosis of the digestive epithelium (DE) and a wider lumen of the digestive tubules (DT) with an accumulation of necrotic epithelial cells (Figure 2B). Some of the epithelium was detached from the digestive gland (DG), and the whole glandular tubules had been eroded, meaning the epithelial cells were severely vacuolated (Figure 2C). Moreover, Gram-negative bacteria were observed in the connective tissue surrounding the digestive tubule and gland (Figure 2D); therefore, the potential pathogenetic bacteria were further isolated.

### 3.2. Isolation and Identification of Bacterial Pathogens in Diseased S. broughtonii

**Figure 3:**
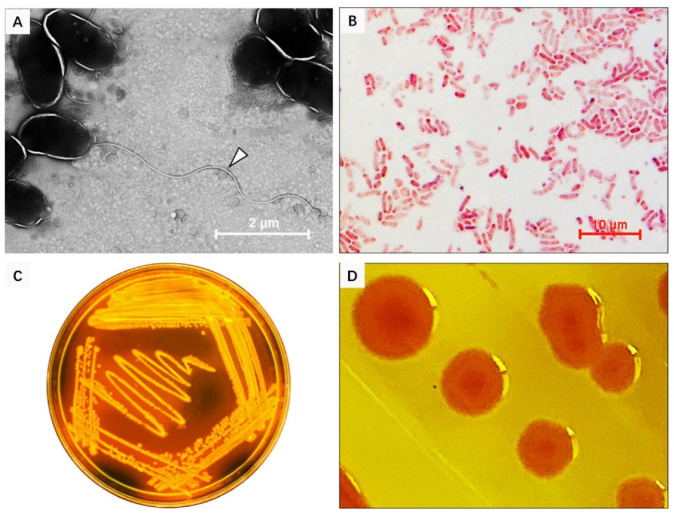
Morphological characteristics of *V. kanaloae* strain SbA1-1. (**A**) The morphological structure of SbA1-1, white arrow indicates the flagellum. SbA1-1 grew in liquid, was negatively stained with 2% phosphotungstic acid, and examined under TEM. Magnification, ×5000. (**B**) The characteristics of SbA1-1 after Gram staining. SbA1-1 shows red staining with a curved rod shape. (**C**,**D**) Colonial morphology and color of SbA1-1 on Thiosulfate Citrate bile salts sucrose (TCBS) agar.

**Figure 4:**
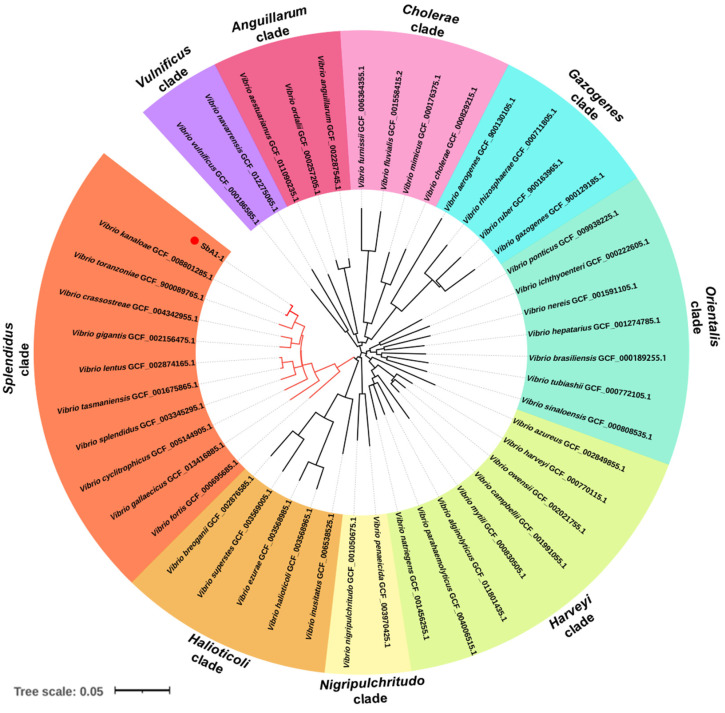
Phylogenetic tree of bacteria (*V. kanaloae* strain SbA1-1) based on concatenated sequences of *atpA*, *mreB*, *pyrH*, *recA* and *rpoA* genes (4958 nt) using the NJ method and visualized in iTOL v6.1.1. (https://itol.embl.de/, accessed on 26 March 2021). The colored sectors indicate distinct *Vibrio* clades. The scale bar corresponds to 0.05 substitutions per site.

The 16S rDNA sequence analysis of 200 isolated bacterial colonies from natural diseased ark clams, *S. broughtonii*, revealed that these colonies constituted 86 ± 0.07% *Vibrio*, 8 ± 0.04% *Pseudoalteromonas*, 4 ± 0.05% *Photobacterium* and 2 ± 0.03% *Tenacibaculum* (Table 2). Within the *Vibrio* group, the bacteria were classified into 13 different species, and *V. kanaloae* was the predominant (39.5 ± 0.15%) species, significantly higher in proportion compared to the others (Table 2). The electronic and light microscopy analysis showed that *V. kanaloae* strain SbA1-1 cells were curved and rod-shaped with one polar flagellum >15 μm in length (Figure 3A). Gram staining revealed that SbA1-1 was Gram-negative, and the colonies were circular on TCBS agar, with entire edges and raised surfaces showing a yellow color (Figure 3B–D). Bacterial taxonomy was further determined via multi-locus sequence analysis (MLSA). A phylogenetic tree generated by a neighbor-joining method based on five housekeeping genes *atpA*-*mreB*-*pyrH*-*recA*-*rpoA* showed nine main clades of vibros: *Splendidus*, *Halioticoli*, *Nigripulchritudo*, *Harveyi*, *Orientalis*, *Gazogenes*, *Cholerae*, *Anguillarum* and *Vulnificus*. The predominant isolate, SbA1-1, was in the *Splendidus* clade and showed the closest relationship to *V. kanaloae* (Figure 4).

### 3.3. Growth Kinetics and Pathogenicity of V. kanaloae Strain SbA1-1 at Low (15 °C) and High (25 °C) Temperatures

To simulate an iron-limited environment, as observed in the host, the *V. kanaloae* strain SbA1-1 was cultured in 2216E medium with different concentrations of DP at 15 °C and 25 °C. Based on the changes in OD600 values, we found that the inhibitory effect of DP on the growth of SbA1-1 was dose-dependent (Figure S2). When the concentration of DP was increased to 160 μM, the growth of SbA1-1 displayed a significant reduction at 15 °C and 25 °C, with a decrease of 33.4% and 26.5% at 48 h, respectively. In the presence of 640 μM DP, the growth of SbA1-1 was completely inhibited at 15 °C and 25 °C, thus the DP MIC to *V. kanaloae* was approximately 640 μM at both temperatures (Figure S2). Due to its significant inhibition and higher bacterial concentration, 160 μM DP was selected to simulate an iron-limiting microenvironment in vivo.

**Figure 5:**
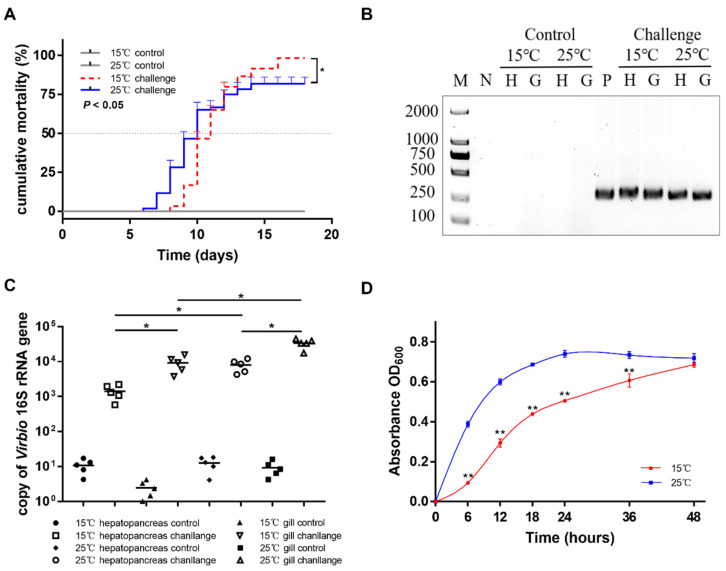
Pathogenicity of SbA1-1 to ark clams. (**A**) Cumulative mortality of ark clams challenged with SbA1-1. Ark clams in the challenge group were immersed in SbA1-1 (1 × 10^5^ CFU/mL) at 15 °C and 25 °C. * *p* < 0.05. (**B**) Detection of challenged ark clams by *V. kanaloae*-specific nested PCR. M: molecular marker; N: negative control; P: positive control; H: hepatopancreas; G: gill. (**C**) The abundance of *Vibrio* in hepatopancreas and gill tissue after SbA1-1 infection in ark clams. ** *p* < 0.01; * *p* < 0.05. (**D**) Growth dynamics of SbA1-1. Growth was assayed in DPM medium by measuring OD_600_ for 48 h at 15 °C and 25 °C. ** *p* < 0.01; * *p* < 0.05.

**Figure 6:**
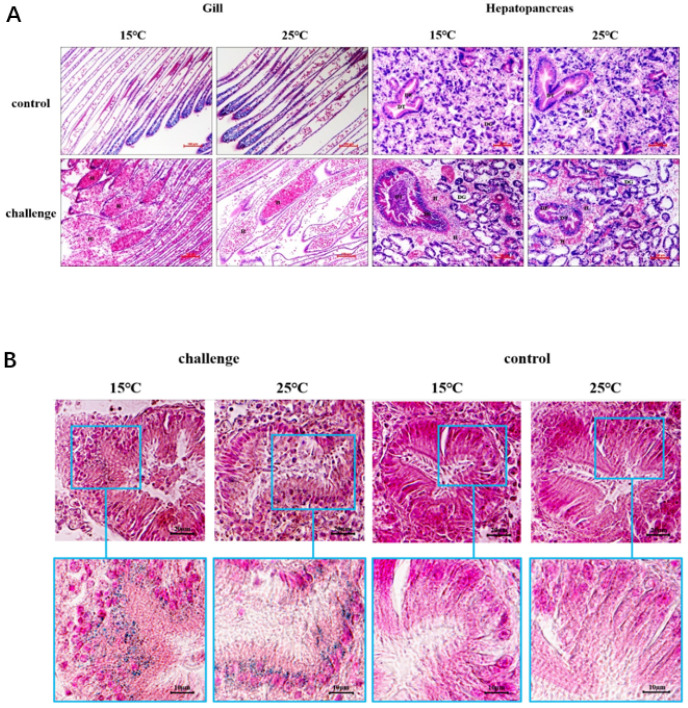
Histopathological changes and iron deposition in ark clam tissues after SbA1-1 infection. (**A**) Histopathological changes in gill and hepatopancreas tissues. Bar = 100 μm. (**B**) Iron deposition in the hepatopancreas showed by Prussian blue stain. H, hemocyte; DE, digestive epithelium; DT, digestive tubule; DG, digestive gland.

To assess the pathogenicity of the *V. kanaloae* strain SbA1-1 at different temperature conditions (15 °C and 25 °C), the mortality of ark clams was compared among groups after the challenge. In the challenge groups, the mortality of ark clams at 15 °C was significantly higher (*p* < 0.05) than at 25 °C, up to 98% on the 16th day after immersion. Additionally, when the ark clam was challenged at 25 °C, 81% mortality was reached after 16 days until the end of the experiment (Figure 5A). Dead ark clams in the 15 °C and 25 °C challenge groups exhibited symptoms that were identical to the natural diseased ark clams: gill erosion, congested gill filaments with broken frontal cells, thin foot, engorged hepatopancreas with extensive necrosis of the digestive epithelium, and a slow response to external stimuli (Figure 1C,D and Figure 6A). Prominent lesions were not observed in the other tissues, including the adductor muscle and mantle (Figure S3). In addition, iron deposition was observed in the submucosa of the hepatopancreatic digestive tube lumen of dead ark clams (Figure 6B). A nested PCR assay specific to *V. kanaloae* was developed, which did not cross-react with 12 non- *V. kanaloae* bacteria isolated from shellfish and the genomic DNA of a healthy ark clam (Figure S4). Using a specific nested PCR assay for the tissue-distribution detection of *V. kanaloae*, *V. kanaloae* could be detected in the gills and hepatopancreas of 10 randomly selected dead ark clams maintained at each temperature (Figure 5B). Simultaneously, the total *Vibrio* load in the gills and hepatopancreas of dead ark clams was significantly increased. *Vibrio* numbers were lower in the hepatopancreas than in the gills and were significantly higher in both tissues at the high temperature compared to the low temperature (Figure 5C). The above results indicated that SbA1-1, which grew slowly when incubated at 15 °C (Figure 5D), showed an increase in pathogenicity at the same temperature compared to 25 °C.

### 3.4. Temperature Effect on the Hemolytic Activity and Siderophore Production in V. kanaloae Strain SbA1-1

**Figure 7:**
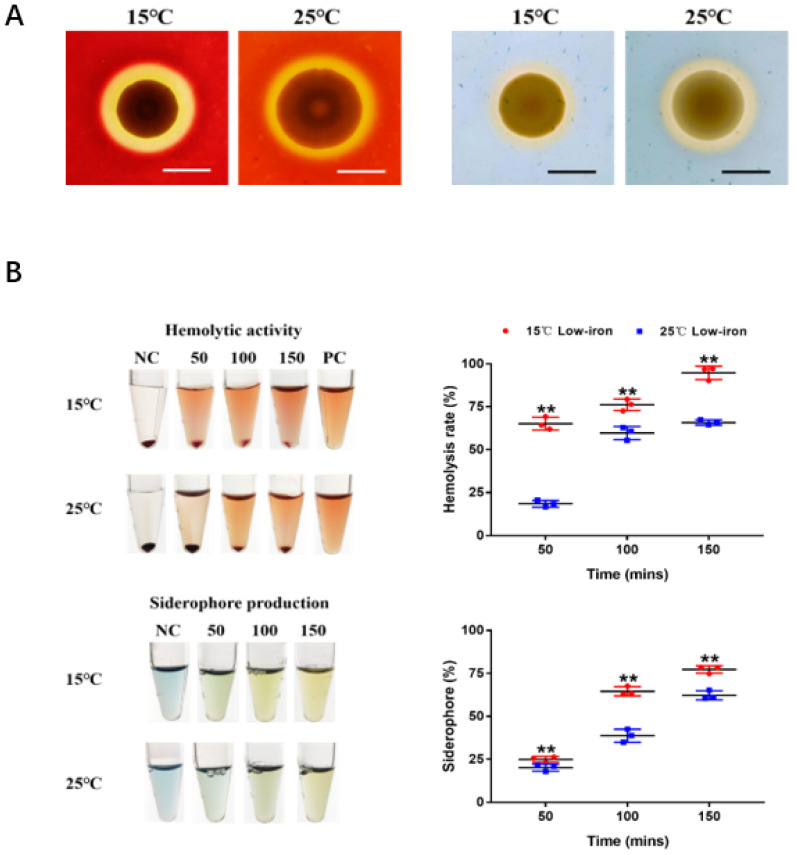
Effect of temperature on hemolytic activity and siderophore production of SbA1-1. (**A**) Hemolytic activity (left) and siderophore production (right) of SbA1-1 at low (15 °C) and high (25 °C) temperatures. Bar = 1 cm. (**B**) Quantification of hemolytic activity and siderophore production by SbA1-1 at different temperatures. Tubes show the color change of hemolytic and CAS reaction at different time points under 15 °C and 25 °C temperature conditions. The corresponding scatter plots show the value changes. ** *p* < 0.01.

The hemolytic activity and siderophore production of the *V. kanaloae* strain SbA1-1 were initially observed on blood agar plates and CAS agar plates at 15 °C and 25 °C. SbA1-1 produced a higher translucent hemolysis halo at 15 °C compared to 25 °C, while SbA1-1 formed a larger yellow–orange halo on CAS plates at 25 °C (Figure 7A). The quantification assays indicated that the hemolytic activity and siderophores productivity of SbA1-1 were significantly enhanced at the low growth temperature (*p* < 0.01) (Figure 7B). When SbA1-1 was incubated at the low temperature (15 °C) after 50, 100 and 150 min, the hemolysis rate increased to 3.47 ± 0.30, 1.28 ± 0.06 and 1.44 ± 0.02-fold, respectively, compared to that at the high temperature (25 °C). Meanwhile, the percentage of siderophores at the low temperature was up-regulated to 1.27 ± 0.11, 1.69 ± 0.01, and 1.22; ± 0.05-fold at 50, 100 and 150 min, respectively.

## 4. Discussion

In winter 2020, a diseased ark clam population was collected that showed consistent symptoms of previously described bacteriosis [4]. Furthermore, the presence of bacteria was observed in the connective tissue of the hepatopancreas by microscopic examination. This suggested that this disease could be caused by bacterial infection. A dominant and potential bacterial pathogen was isolated and the identical symptoms, as natural incidences, were reproduced by artificial infection. Meanwhile, the temperature effect on its pathogenicity was further analyzed.

*Vibrio* is the primary pathogen affecting all life stages of many marine molluscs including larvae, juveniles, and adults [13,23]. In this study, *Vibrio* spp. were the largest bacterial group, accounting for 86 ± 0.07% of the total bacteria isolated from ark clam lesions, and *V. kanaloae* was the predominant and most common species compared to the others. *V. kanaloae* belongs to the clade of ***the** V. splendidus* evolutionary branch that is ubiquitous in aquatic environments [23] and has a pathogenic potential for aquatics, including mollusks (*Crassostrea gigas*) [57], crustaceans (*Artemia* nauplii) and fish (*Oncorhynchus mykiss*, Walbaum) [58]. In contrast to the symptoms caused by *V. kanaloae* in *C. gigas* and *O.mykiss*, the symptoms in ark clams are different. The main symptom of *V. kanaloae* in *C. gigas* and *O.mykiss* is muscle tissue damage [57]. However, *V. kanaloae* could induce severe gill hyperemia and erosion in ark clams at either high or low temperatures. Gills play an essential role as natural barriers in removing and eliminating potential pathogens [59]. Combining histopathological changes with tissue *Vibrio* loads, gills erosion might provide an entrance for *V. kanaloae* to disseminate through the open circulatory system to other tissues of the ark clams [60]. Severe hyperemia was found in the hepatopancreas after *V. kanaloae* infection, which was considered as a typical mollusk inflammatory response to pathogen invasion and tissue damage [61,62]. In the ark clam, both hepatopancreas and hemocytes serve as the main iron storage tissue [61]; the hepatopancreas hyperemia appears to provide a desirable site for *V. kanaloae* growth and colonization [63,64]. The increased iron content and *Vibrio* quantity in the hepatopancreas support the aforementioned conjecture, implying that the hepatopancreas was a susceptible tissue for *V. kanaloae* and the successful colonization of the hepatopancreas by *Vibrio* might be the major cause of ark clam mortality.

Temperature is a major driver of vibriosis outbreaks [32,65]. Aquatic mortality caused by *Vibrio* pathogens is usually associated with elevated temperatures [4,12,29,66,67,68]. However, there are also several marine vibrios capable of copiously growing over a wide range of temperatures and remaining pathogenic at low temperatures [12,28,32]. Bacteria accommodate temperature variations by modulating not only the whole-cell enzyme activity but also metabolic and virulent genes expression [28,69,70]. Our results showed that *V. kanaloae* possessed a wide range of temperature adaptability. Although the growth ratio of *V. kanaloae* was reduced by the low temperature, *V. kanaloae* maintained a strong pathogenicity, even slightly higher than the high temperature. This phenomenon was also present in other pathogenic *Vibrio* species, such as *V. anguillarum* and *V. salmonicida*, which possessed a high pathogenicity at low temperatures and were accompanied by altered iron metabolism [32,33,71].

The iron uptake process of bacterial pathogens was often associated with their pathogenic process [42,72,73]. Hemolysin and siderophore are critical virulence factors for bacterial pathogen colonization and the establishment of infection [33,42,74,75]. The temperature-regulated production of hemolysin and siderophore has been identified in several bacterial genera. For example, *V. anguillarum* favors the synthesis of piscibactin siderophore at low temperatures, and the hemolytic activity of *V. splendidus* decreased as temperature increased [18,33]. In the present study, *V. kanaloae* could produce hemolysin and siderophore and was regulated by temperature. Additionally, hemolysin could trigger cell membrane rupture leading to tissue damage [42,76] and siderophores could hijack host available iron [34]. The enhanced productivity of the two virulence factors associated with iron uptake at 15 °C would partially explain the higher pathogenicity of *V. kanaloae* at the low temperature.

Therefore, the outbreaks of vibriosis might be caused by *V. kanaloae* during cold seasons, and this should be given more research attention. The nested PCR assay developed in this study is proven to be available for the routine diagnosis and monitoring of *V. kanaloae* in cultured ark clams.

## 5. Conclusions

Overall, a pathogenic *V. kanaloae* strain SbA1-1 was originally isolated and identified from a diseased ark clam population during cold seasons. Our results demonstrated that SbA1-1 was highly pathogenic to ark clams at both high (25 °C) and low (15 °C) temperatures and exhibited a stronger hemolytic activity and siderophores productivity at 15 °C. Altogether, these findings suggest that *V. kanaloae* should be monitored among cultured ark clams, especially during cold seasons.

## Figures and Tables

**Table 1:** PCR primers used in this study.

Primer Name	Product	Primer Sequence (5′-3′)	References/GenBank Accession Number
OsHV-1 quantification primers			[43]
BF		GTCGCATCTTTGGATTTAACAA	
B4		ACTGGGATCCGACTGACAAC	
BP		FAM-TGCCCCTGTCATCTTGAGGTATAGACAATC-BHQ	
*Vibrio* housekeeping gene primers			
27F	16S rDNA	AGAGTTTGATCCTGGCTCAG	[44]
1492R		GGTTACCTTGTTACGACTT	
atpA-F	ATP synthase alpha subunit	CTDAATTCHACNGAAATYAGYG	[46]
atpA-R		TTACCARGWYTGGGTTGC	
mreB-F	rod shaping protein B subunit	ACTTCGTGGCATGTTTTC	[47]
mreB-R		CCGTGCATATCGATCATTTC	
pyrH-F	uridylate kinase	ATGASNACBAAYCCWAAACC	[48]
pyrH-R		GTRAABGCNGMYARRTCCA	
recA-F	recombinase A	TGARAARCARTTYGGTAAAGG	[48]
recA-R		TCRCCNTTRTAGCTRTACC	
rpoA-F	RNA polymerase alpha subunit	ATGCAGGGTTCTGTDACAG	[49]
rpoA-R		GHGGCCARTTTTCHARRCGC	
Total *Vibrio* quantification primers			
Vib1	16S rDNA	GGCGTAAAGCGCATGCAGGT	[50]
Vib2		GAAATTCTACCCCCCTCTACAG	
Specific nested PCR primers			
Vkan-F1	IucA/IucC family siderophore biosynthesis protein	TCGTTTTGATTTTGACTTAGGACGC	QPK06640.1
Vkan-R1		CTTGAGGTGATACACCTGAGCGTTC	
Vkan-F2	IucA/IucC family siderophore biosynthesis protein	GTCGATATTCAGATCAGGAGTCGTC	QPK06640.1
Vkan-R2		CCTTGTTAAAAGCAAATTTAGGGTG	

**Table 2:** Bacteria isolated from the diseased *S. broughtonii*.

Bacterial Names	Frequency of Occurrence ^a^	Number	Proportion (%) ^b^
***Vibrio***		**172**	**86 ± 0.07**
*V. fischeri*	2/10	3	1.5 ± 0.03
*V. penaeicida*	3/10	3	1.5 ± 0.02
*V. atlanticus*	3/10	4	2.0 ± 0.03
*V. crassostreae*	4/10	4	2.0 ± 0.03
*V. splendidus*	3/10	4	2.0 ± 0.03
*V. lentus*	4/10	5	2.5 ± 0.04
*V. gigantis*	5/10	6	3.0 ± 0.03
*V. crassostreae*	3/10	7	3.5 ± 0.05
*V. alginolyticus*	5/10	9	4.5 ± 0.05
*V. cyclitrophicus*	5/10	12	6.0 ± 0.07
*V. toranzoniae*	5/10	16	8.0 ± 0.09
*V. harveyi*	6/10	20	10.0 ± 0.10
***V. kanaloae* ^c^**	10/10	79	39.5 ± 0.15
***Pseudoalteromonas***		**16**	**8 ± 0.04**
*P. atlantica*	4/10	4	2.0 ± 0.03
*P. issachenkonii*	2/10	4	2.0 ± 0.04
*P. tetraodonis*	2/10	4	2.0 ± 0.04
*P. elyakovii*	2/10	4	2.0 ± 0.04
***Photobacterium***		**8**	**4 ± 0.05**
*P. swingsii*	5/10	8	4.0 ± 0.05
***Tenacibaculum***		**4**	**2 ± 0.03**
*T. lutimaris*	3/10	4	2.0 ± 0.03
Total		200	

^a^ Occurrence frequency of bacteria in 10 individuals. ^b^ Mean of the proportion of bacteria to total isolated bacteria in each individual. ^c^ Bold highlight the dominated strains.

## Data Availability

Not applicable.

## Supplementary Materials

The following are available online at https://www.mdpi.com/article/10.3390/microorganisms9102161/s1. Figure S1. OsHV-1 detection in natural diseased ark clams. Figure S2. Growth of SbA1-1 in 2216E medium supplemented with different concentrations of DP at 15 °C (A) and 25 °C (B). Figure S3. Histology of adductor muscle and mantle tissues of ark clams after SbA1-1 infection at 15 °C and 25 °C. Figure S4. Specificity analysis of the nested PCR method.
