# Supplementary material for: Isolation and Characterization of Vibrio kanaloae as a Major Pathogen Associated with Mass Mortalities of Ark Clam, Scapharca broughtonii, in Cold Season
Source: Microorganisms. 2021 Oct 16;9(10):2161. doi: 10.3390/microorganisms9102161 (PMC8541523; doi:10.3390/microorganisms9102161)
Supplement: Supplementary file 1 [file microorganisms-09-02161-s001.zip › microorganisms-1389494.supplementary.pdf]

Supplementary Materials

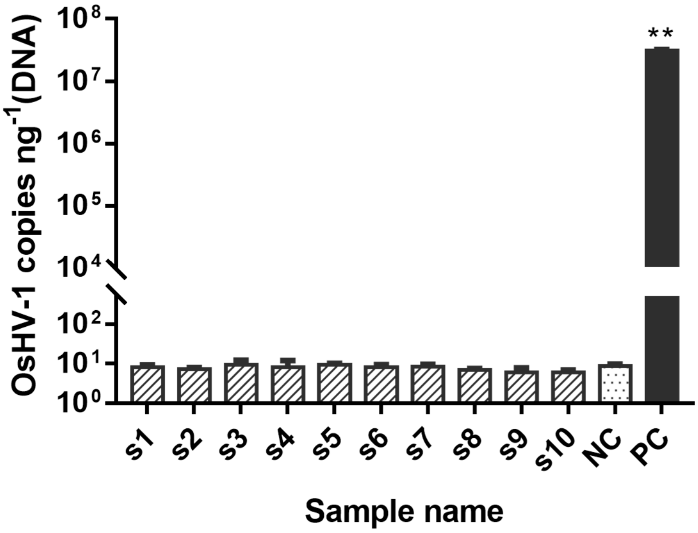

Figure S1. OsHV-1 detection in natural diseased ark clams. The load of OsHV-1 was quantified by qPCR and expressed as viral genomic copies per ng of total tissue DNA. **$p < 0.01$.

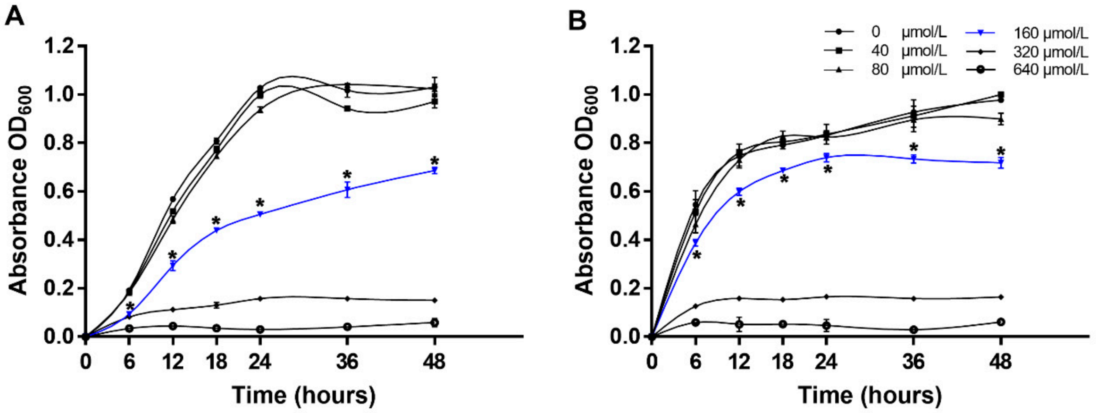

Figure S2. Growth of SbA1-1 in 2216E medium supplemented with different concentrations of DP at 15 °C (**A**) and 25 °C (**B**).

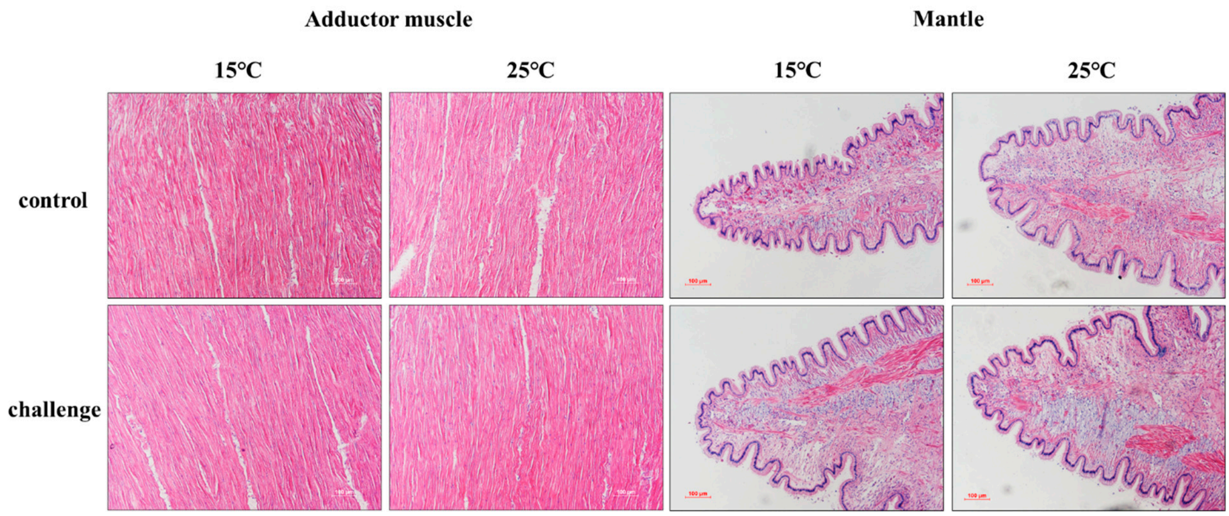

Figure S3. Histology of adductor muscle and mantle tissues of ark clams after SbA1-1 infection at 15 °C and 25 °C. Bar = 100 μm.

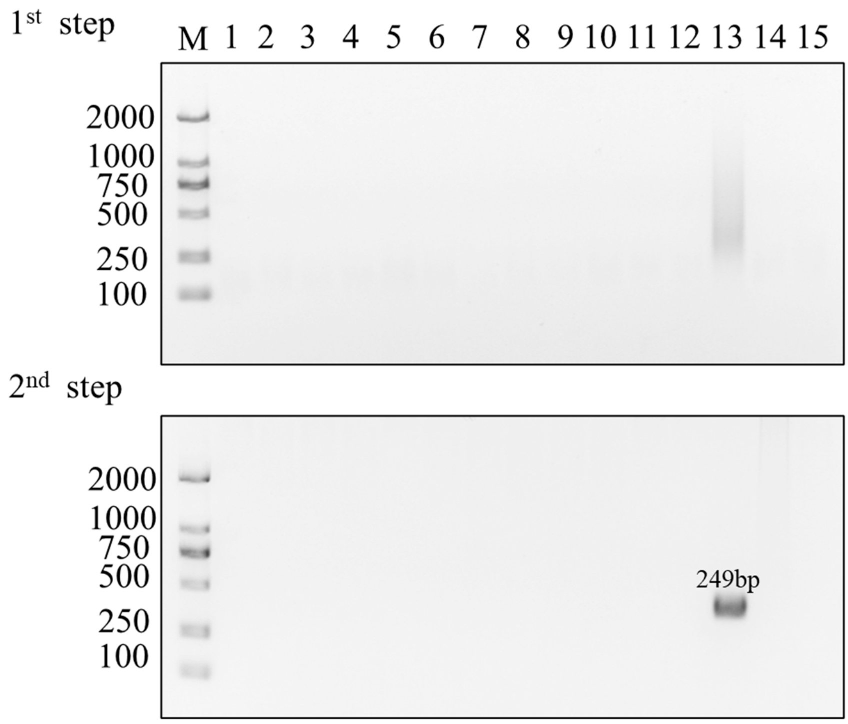

Figure S4. Specificity analysis of the nested PCR method. M, Molecular marker; Lanes 1-13: PCR amplified products with DNA templates extracted from *V.splendidus*, *V.toranzoniae*, *V. harveyi*, *V. crassostreae*, *V.gigantis*, *V.cyclitrophicus*, *V.alginolyticus*, *Pseudoalteromonas phenolica*, *Pseudoalteromonas atlantica*, *Psychrobacter marincola*, *Photobacterium swingsii*, *Tenacibaculum lutimaris* and *V. kanaloae*, respectively; lane 14: PCR amplified products with DNA template sample extracted from healthy ark clams; and lane 15: blank control.
